# Osteoimmunology: A Link between Gastrointestinal Diseases and Skeletal Health in Chickens

**DOI:** 10.3390/ani13111816

**Published:** 2023-05-30

**Authors:** Milan Kumar Sharma, Prafulla Regmi, Todd Applegate, Lilong Chai, Woo Kyun Kim

**Affiliations:** Department of Poultry Science, University of Georgia, Athens, GA 30602, USA; milan.sharma@uga.edu (M.K.S.); pregmi@uga.edu (P.R.); applegt@uga.edu (T.A.); lchai@uga.edu (L.C.)

**Keywords:** osteoimmunology, coccidiosis, necrotic enteritis, skeletal health

## Abstract

**Simple Summary:**

Gastrointestinal diseases in poultry are more significant because of high economic loss due to production loss, reduced feed efficiency, mortality, compromising bird welfare, and sometimes zoonotic importance. With the removal of antibiotic growth promoters in the diet, gastrointestinal disease incidences have grown in recent years. Gastrointestinal diseases have been shown to affect bone growth negatively; however, possible mechanisms have yet to be confirmed in chickens.

**Abstract:**

Bone serves as a multifunctional organ in avian species, giving structural integrity to the body, aiding locomotion and flight, regulating mineral homeostasis, and supplementing calcium for eggshell formation. Furthermore, immune cells originate and reside in the bone marrow, sharing a milieu with bone cells, indicating a potential interaction in functions. In avian species, the prevalence of gastrointestinal diseases can alter the growth and the immune response, which costs a great fortune to the poultry industry. Previous studies have shown that coccidiosis and necrotic enteritis can dramatically reduce bone quality as well. However, possible mechanisms on how bone quality is influenced by these disease conditions have not yet been completely understood, other than the reduced feed intake. On the other hand, several mediators of the immune response, such as chemokines and cytokines, play a vital role in the differentiation and activation of osteoclasts responsible for bone resorption and osteoblasts for bone formation. In the case of *Eimeria* spp./*Clostridium perfringens* coinfection, these mediators are upregulated. One possible mechanism for accelerated bone loss after gastrointestinal illnesses might be immune-mediated osteoclastogenesis via cytokines-RANKL-mediated pathways. This review article thus focuses on osteoimmunological pathways and the interaction between host immune responses and bone biology in gastrointestinal diseases like coccidiosis and necrotic enteritis affecting skeletal health.

## 1. Introduction

The avian skeletal system is a complex organ with multiple functions providing structural integrity to the body and maintaining homeostasis of minerals such as calcium and phosphorus [1,2,3]. Furthermore, bone marrow is the primary site for hematopoiesis, harboring hematopoietic stem cells, and mature immune cells (B cells, neutrophils, macrophages, and T cells), defending the body against invading pathogens [4,5,6,7]. Immune cells originate and some of them reside within the bone marrow and share the same microenvironment as bone cells, indicating a potential for interactions. The interaction between the bone and immune cells has already been described wherein osteoblasts are a regulator of the hematopoietic stem cells and osteoclast having the same origin as the immune cells, i.e., macrophages and dendritic cells [4,6,7]. Moreover, several mediators of the immune response, such as chemokines and cytokines, play a vital role in the differentiation and activation of osteoclasts and osteoblasts [4,5,6,7]. Since skeletal and immune systems are closely associated with each other, immune cells, chemokines, and cytokines may play a critical role in regulating bone remodeling during disease conditions in avian species. Osteoimmunology, which studies possible interactions between the immune system and bone biology and how they cross-communicate to modulate their respective functions under one umbrella, might be a link in describing bone loss in chickens when infected with gastrointestinal diseases [4,6,8].

Recently, skeletal disorders in broilers and laying hens have resurfaced with advancements in genetics and modern production systems. The selective breeding for higher growth rates in broilers has led to the development of immature skeletal systems unable to withstand the rapidly increasing body mass, resulting in lameness and bone disorders [9,10]. Similarly, the selection of laying hens for early sexual maturity, small body conformation, and maximum egg production has put significant strain on their skeletal systems, as medullary bone serves as a labile source of calcium for eggshell production, potentially contributing to bone disorders [1,2,11,12,13]. Moreover, the intensive production systems utilized in modern poultry farming involve high stocking densities, which provide ideal conditions for transmitting gastrointestinal diseases such as coccidiosis, necrotic enteritis, salmonellosis, etc. [14,15,16]. These gastrointestinal diseases have a substantial economic impact on the poultry industry primarily due to a reduction in body weight and worsened feed conversion ratio [17,18,19,20]. The estimated global loss due to coccidiosis is around 15 billion dollars, whereas that of necrotic enteritis is 6 billion dollars to the poultry industry [16,21]. These gastrointestinal diseases also cause severe inflammation, affecting birds’ bone growth and remodeling. The negative impacts of gastrointestinal diseases and their effect on bone in poultry have already been observed in broilers and laying hens [8,22,23,24,25,26,27,28]. The economic losses associated with bone disorders resulting from gastrointestinal diseases and the possible mechanism other than reduced feed intake for bone loss in these conditions have not been fully understood. Therefore, it is crucial to understand the complex relationship between gastrointestinal diseases, immune response, and skeletal disorders in poultry to develop effective intervention strategies for controlling and preventing these conditions, ultimately promoting the health and welfare of poultry in modern production systems.

In this review, we connect gastrointestinal diseases with skeletal disorders, focusing on the immune responses during and after coccidiosis and necrotic enteritis infections. Understanding this link might lead to a better understanding of the underlying mechanisms and potential therapeutic strategies to control gastrointestinal and skeletal disorders in poultry. Ultimately, understanding the complex relationship between the immune and skeletal systems in avian species might be of great importance for promoting their health and welfare while maintaining their maximum genetic potential.

## 2. Bone Biology of Broilers and Layers

### 2.1. Long Bone Growth

Avian skeletal development begins as early as the embryonic stage and reaches maturity during rearing [1,3,29]. During post-hatch growth, the cartilaginous template from the embryonic stage is replaced by the process of endochondral ossification [30,31]. In the early phase, a thin bony collar is gradually formed around the diaphysis of the cartilaginous bone template through perichondral ossification, allowing lengthwise growth. Lengthwise growth takes place at the primary ossification site through endochondral ossification [1,30]. At the end of the cartilage template or the epiphyseal growth plate, chondrocytes transform into proliferative chondrocytes and are arranged in columns within an extracellular matrix containing type II collagen. Following proliferation, these chondrocytes differentiate into hypertrophic chondrocytes, which secrete type X collagen and later undergo apoptosis and resorbed. Other than type II and type X collagen, chondrocytes also secrete organic matrix containing proteoglycans, glycoproteins, and growth factors, which regulate the further development of chondrocytes [1]. The lengthwise growth of the bone starts at the primary ossification site where chondrocytes lay down the cartilage. In contrast, chondroclast reabsorbs the extracellular matrix, while alkaline phosphatase secreted by chondrocytes and osteoblasts initiates bone calcification [1].

Osteoblasts, derived from mesenchymal stem cells, secrete type I collagen and raise the concentration of Ca^++^ and PO_4_^3−^, forming calcium hydroxyapatite crystals around the matrix [1]. During bone elongation, cartilage is resorbed, resulting in the formation of marrow spaces. The bone is resorbed and remodeled by a collaborative effort of osteoclasts and osteoblasts ultimately resulting in the formation of the trabecular bone in the marrow space [1,2]. Bone modeling allows for longitudinal and periosteal growth during early growth stages. However, hormonal changes after sexual maturity in egg-laying hens lead to the cessation of cortical and trabecular bone formation as well as longitudinal growth of long bones [1,2,12,32]. As hens sexually mature, circulating levels of estrogen significantly increase, which stimulates the function of osteoblasts for the formation of medullary bone at the expense of cortical and trabecular bones, while inhibiting osteoclastic bone resorption. Towards the end of the lay, when circulating estrogen declines, the function of osteoblast is reversed towards the structural bone formation [1,2,32]. Nevertheless, flat bones such as keel bones and ribs continue to ossify even after hens enter laying to provide structural support during egg-laying and maintain their strength. Before the first oviposition, the inner diameter of the long bones increases by approximately 20% through intramembranous ossification and enables the deposition of medullary bone along the marrow cavity. Osteoblasts create a network of immature osteoid along the outer surface of the periosteum, which eventually undergoes calcification to form cortical bones, while osteoclasts resorb bone on the endosteal surface, resulting in the widening of the bone [1]. At sexual maturity, the estrogen surge alters osteoblasts’ function toward medullary bone formation [1,2,32]. Medullary bone is a secondary bone structure with a highly woven texture deposited in the marrow cavities and on the trabecular surfaces. During the pre-lay stage of the growth, medullary bone in marrow space accumulates rapidly and continues to deposit for the rest of the laying period [1,2,32,33]. In the meantime, blood supply plays a crucial role in the development and growth of bones in chickens, and avian vasculature penetrates deeper into the growth plate to support their fast growth rate [34]. The avian blood vasculature not only triggers the calcification of the cartilage matrix and formation of bone marrow, but also recruits osteoblast and osteoclast precursor cells to bone remodeling sites. It also supplies nutrients for collagen and bone formation, removes the metabolites from bone resorption, delivers systemic hormones and precursor cells, and provides angiogenic and angiocrine signals regulating bone formation, resorption, and remodeling [34,35].

### 2.2. Bone Remodeling

Bone remodeling is a dynamic and continuous process to maintain skeletal integrity and functionality under various stressors including physiological, hormonal, or mechanical [1,6,32,33]. This dynamic process involves the resorption and formation of bones by osteoclast and osteoblast, respectively. Osteoclasts are multinucleated bone-resorbing cells derived from monocyte/macrophage cells of hematopoietic origin [5,36]. They are more abundant in bone surfaces and are engaged in bone degradation. Whereas osteoblasts are bone-forming cells that originate from mesenchymal stem cells residing in the bone matrix of the bone marrow. These metabolically active cells synthesize the collagenous and non-collagenous bone matrix and facilitate calcification [1,5,6]. During this process, osteoblasts are trapped in the bone matrix and are converted to osteocytes, which regulate the pace of bone modeling and remodeling [5,6,37]. In fast-growing broilers, bone remodeling occurs rapidly due to the fast growth rate and high metabolic demand of bone tissue [29]. Bone remodeling occurs in both types of structural bone tissue, cortical and trabecular. Cortical bone is denser and provides structural support to the skeleton, while the trabecular bone is more porous and plays a role in mineral metabolism and load-bearing function [1,2]. Trabecular bone, due to its larger surface area and higher metabolic activity compared to cortical bone, is more susceptible to higher rates of bone remodeling [8,22]. However, medullary bone in laying hens is highly vascularized and plays a crucial role in maintaining calcium homeostasis [1,2,13,32]. Furthermore, it serves as a labile source of calcium for eggshell formation, and the process of its deposition and resorption occurs continuously throughout the laying period. Therefore, medullary bone remodeling in egg-laying chickens is a significant physiological process that allows a hen to meet the increased demand for calcium during egg production.

**Figure 1:**
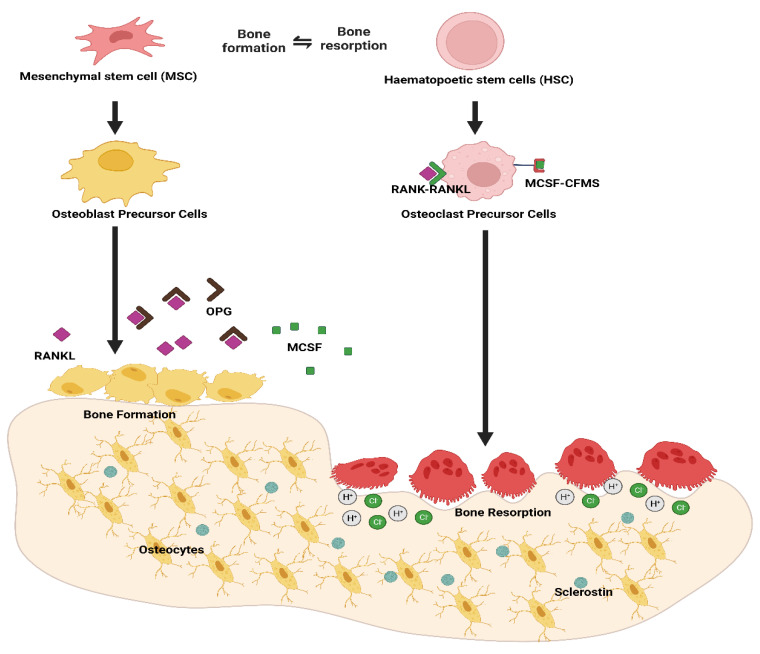
The mechanism of mesenchymal and hematopoietic cell differentiation into osteoblast and osteoclast, along with the bone remodeling process. RANKL: Receptor activator of nuclear factor κB ligand, RANK: Receptor activator of nuclear factor κB, OPG: Osteoprotegerin, MCSF: Macrophage colony-stimulating factor, CFMS: Colony stimulating factor receptor, H^+^: Hydrogen ion, Cl^−^: chloride ion.

The dynamic process of bone remodeling in chickens is tightly regulated by the osteoclastogenesis, and osteoblastogenesis at the cellular level and is initiated by the change in the secretion of hormones for calcium homeostasis (parathyroid, calcitriol, calcitonin, or estrogen hormones), the status of lay, mechanical stress, or immune responses. Bone remodeling is completed in three different phases: (i) initiation of resorption by osteoclasts, (ii) transitioning from resorption to the formation of new bone (reversal), and (iii) formation of new bones [6,7,38]. A change in calcium homeostasis, mechanical stress, or damage to the bone stimulates osteocytes and osteoblasts to recruit osteoclast precursor cells in bone surfaces. An increase in the expression of receptor activator of nuclear factor kappa-Β ligand (RANKL), decreased in osteoprotegerin (OPG) from osteoblast and increased in sclerostin from osteocytes, stimulates RANKL-mediated osteoclastogenesis [37,39,40,41,42,43]. These multinucleated osteoclasts then degrade and resorb the mineralized matrix by producing collagenolytic enzymes (Cathepsin K, Tartrate resistant acid phosphatase) and hydrochloric acids and undergo apoptosis [44]. The resorption of mineralized bones is then followed by the reversal phase, where the mononuclear cells remove the remaining collagen [45]. Furthermore, the increased concentration of insulin-like growth factors (I, II) and transforming growth factors B recruit mesenchymal stem cells to bone resorption sites and differentiate into osteoblasts [46]. The newly differentiated osteoblasts lay down the collagenous bone matrix in the resorbed sites called osteoid, which later calcifies. The bone remodeling concludes once the equilibrium is achieved, and then osteoblasts either undergo apoptosis or embed themselves in the bone matrix and differentiate into osteocytes [43,47]. The detailed process of bone remodeling is visually illustrated in Figure 1.

## 3. Host-Immune Response in Coccidiosis and Necrotic Enteritis

Coccidiosis and necrotic enteritis have the most significant impact on the poultry industry, with more than 20 billion dollars in loss globally per annum [16,21]. Coccidiosis is caused by several species of *Eimeria* (*Eimeria acervulina*, *Eimeria brunetti*, *Eimeria maxima*, *Eimeria mitvai*, *Eimeria necatrix*, *Eimeria mitis*, *Eimeria praecox*, *Eimeria tenella*, *Eimeria hagani*) either alone or in combination [15,19,48,49]. Necrotic enteritis is a highly prevalent gastrointestinal disease in poultry, caused by *Clostridium perfringens* strains expressing NetB toxins and, most of the time, it is secondary to other predisposing factors [50,51,52]. Coccidiosis is one of the major predisposing factors other than high dietary proteins, dysbiosis, or enteric infections altering the immune status of birds and diets, increasing the passage rate of intestinal content [50,52,53]. Coccidiosis and necrotic enteritis affect most of the gastrointestinal tract by disrupting the gastrointestinal barrier, causing severe acute and chronic inflammation of the gastrointestinal lining, tissue necrosis, and reducing the development of immune organs [17,18,19,54,55,56].

The gut-associated lymphoid tissues (GALT) serve as the body’s first line of defense against infection, disease development, and other threats by eradicating infectious pathogens at an early stage [57,58,59]. Pattern recognition receptors, such as Toll-like receptors, are well adapted to the intestinal epithelial cell barrier for microbial sensing through a microbial-associated molecular pattern. GALT functions as antigen recognition and presentation, followed by the release of intestinal antibodies and activation of the cell-mediated immune response in chickens when infected with gastrointestinal diseases [50,52,59,60]. Following this, activated B and T cells move to the lamina propria, where immune responses are put into action [58,59,61,62,63]. Adaptive immunity depends on the T and B cells found in the lamina propria. The immune system reacts swiftly to infection as early as 3 h, mainly indicated by an increased concentration of polymorphonuclear leukocytes (primarily heterophils) in the intestinal villi, the antigen’s point of invasion [63].

The gastrointestinal mucosal lining is a part of the intestinal defense mechanism and helps recognize the pathogen and activate innate immune responses. Toll-like receptors recognize the microbial-associated pattern factor in and on microbes and initiate the signal transduction cascade that leads to the transcription of numerous genes involved in the immune response through RANK-mediated pathways. Activation of the antigen-presenting cells (APCs), such as dendritic cells and macrophages, by the pathogens and upregulation of pro-inflammatory cytokines and chemokines by innate immune cells are essential for developing adaptive immunity [50,52,58,59,61,64]. Upon activation, APCs migrate to regional lymphoid organs, where they upregulate the production of pro-inflammatory cytokines, such as IL-1β, IL-6, IL-8, and IL-12, facilitating the activation of naive CD4+ T-cells. Subsequently, these activated T-cells differentiate into various subsets, including Th1, Th2, Treg, and Th17 [62,65,66,67,68,69]. It has also been observed that necrotic enteritis and coccidiosis significantly increase the number of naive CD4+ cells, which is supposed to differentiate into different subsets and cytotoxic T-reg cell (CD8+) of a cell-mediated immune response [51,70].

In *Clostridium perfringens*/*Eimeria* spp. co-infected necrotic enteritis, Th1 cells are activated as part of the adaptive immune response. The cytokines produced by Th1 cells (IFN-γ, IL-2, IL-12, and TNF-α) can help clear the infection by promoting the activation of macrophages and other immune cells that can eliminate the intracellular pathogens [50,58,62,65,69]. Studies have demonstrated that cytokines associated with cell-mediated immunity, such as IFN-γ, IL-2, and TNF-alpha, are upregulated during coccidiosis and necrotic enteritis [17,19,65]. However, IFN-γ and IL-2 were reported to be downregulated in necrotic enteritis to prevent the activation of innate immune system cells and suppress antigen presentation by APCs [71]. These cytokines can promote the activation and proliferation of other immune cells, such as cytotoxic T cells and natural killer cells. Furthermore, Th2 cytokines such as IL-3, IL-4, IL-9, IL-10, and IL-13 are also upregulated during necrotic enteritis as a part of the humoral defense system [50]. Previous studies have suggested that Th2 cytokines such as IL-4, IL-9, IL-10, and IL-13 may play a role in limiting the severity of the disease by promoting tissue repair and limiting inflammation and promoting antigen-specific B cell activation and antibody production. Th17 cells play a crucial role in microbial defense at mucosal surfaces, as they upregulate the production of cytokines IL-17 and IL-22, secretion of antimicrobial peptides, IgA, and the induction of inflammation, all of which contribute to defense against invading pathogens. Furthermore, Th17 cells also aid in the regeneration of intestinal epithelial cells [72,73]. The upregulation of immune response is critical in protecting chickens against various infectious diseases including coccidiosis and necrotic enteritis. These immune responses are not limited to the intestinal mucosa but might also have an influence on skeletal homeostasis altering the skeletal integrity.

## 4. Shift in Gut Microbiome and Immune Response

Diverse microbial communities naturally colonize the GIT of chickens and are an integral part of the intestinal ecosystem alongside the intestinal epithelial lining and immune system [74,75,76,77]. Microbiomes and immune cells residing in the GIT coexist in equilibrium while maintaining the functionality to respond against harmful pathogens. The crosstalk between the intestinal microbiome and immune system, and epithelial cells strengthens their symbiotic relationship. The host immune system regulates the colonization and microbial composition, whereas the interaction between the microbiome helps in the development and functionality of the immune system [74,75,77,78]. It has been observed that gut microbiome plays a significant role in goblet cell development [79], B cell development and activation in lamina propria and Bursa of Fabricius [80], T cell differentiation [81,82] and development of GALT [75,80]. Furthermore, metabolites produced by the gut microbiota such as short-chain fatty acids, indole, tryptamine, vitamins, and bacteriocins were reported to increase the production of anti-inflammatory cytokines by altering inflammasomes thus reducing the harmful inflammation [74,82].

However, a shift in the commensal microbial population was observed after coccidiosis and necrotic enteritis infection. A decrease in segmented filamentous bacterial population was observed in the intestine and ceca of infected birds [83]. These bacterial populations are involved in the differentiation of Th17 cells from naïve CD4+ T cells along with the IgA production [84]. Likewise, a shift in lactic acid-forming bacterial populations (either increased or decreased) was also observed following the infection [83,85,86,87,88,89,90]. These bacterial populations were involved in maintaining tight junction integrity and immunomodulation by stimulating TNF-α, INF-γ, and IL-12 [91]. Similarly, short-chain fatty acids-producing bacterial populations were decreased in chickens following the infection [83,85,86,87,88,89,90].

## 5. Interaction between Immune Response against Gastrointestinal Disorders and Bone Biology

Since mature immune cells (B cells, macrophages, and T cells), hematopoietic stem cells, and bone cells (osteoblasts, osteoclasts, and osteocytes) all coexist in the same milieu (bone marrow), they interact with one another to perform their respective functions [4,6,7]. Osteoclasts originate from hematopoietic stem cells, from which blood cells and other immune cells originate too. Osteoclasts are derived from monocytes/macrophages, which are circulating white blood cells that play a significant role in immune functions. In contrast, osteoblasts are derived from mesenchymal stem cells. Human and animal studies have revealed that several immune cytokines released from activated immune cells influence bone cell activity and remodeling through RANKL during inflammatory conditions [51,52,53,71,72].

**Figure 2:**
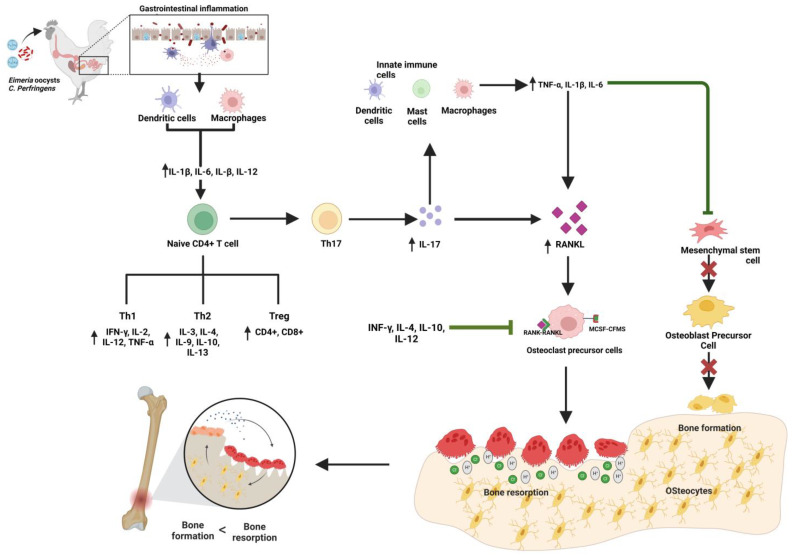
Schematic representation of the interaction between immune responses and bone biology following gastrointestinal disorder (coccidiosis and necrotic enteritis) in chickens. IL-1β: Interleukin 1 β, IL-6: Interleukin 6, IL-12: Interleukin 12, Th1: T helper cells 1 (Cell-mediated immunity), Th2: T helper cells 2 (Humoral immunity), Treg: Regulatory T cells, Th17: T helper cells 17, IFN-γ: Interferon γ, IL-2: Interleukin 2, TNF-α: Tumor necrosis factor α, IL-3: Interleukin 3; IL-4: Interleukin 4, IL-9: Interleukin 9, IL-10: Interleukin 10; IL-13: Interleukin 13; IL-17: Interleukin 17, RANKL: Receptor activator of nuclear factor κB ligand, RANK: Receptor activator of nuclear factor κB, OPG: Osteoprotegerin, MCSF: Macrophage colony-stimulating factor, CFMS: Colony stimulating factor receptor, H^+^: Hydrogen ion, Cl^−^: chloride ion.

The nuclear factor kappa-ligand (RANK) is a common link between the immune and skeletal systems. RANKL, along with macrophage colony-stimulating factor (MCSF), signals the monocytes and macrophages to differentiate into osteoclasts, known as osteoclastogenesis [5,6,41]. Osteoblasts and osteocytes express RANKL under the influence of hormones and physiological factors [37,41,43,47]. Pro-inflammatory cytokines from activated immune T cells, TNF-α, IL1β, and IL-17 intensely expressed the RANKL [42,92,93], which is key to the differentiation of osteoclasts after binding with their receptor RANK from monocyte-osteoclast precursors, and their activity. The OPG extensively regulates the interaction of RANKL with RANK, a decoy receptor for RANKL expressed by osteocytes and osteoblasts [94]. The interaction between the immunological pathways connecting to the bone remodeling is visually illustrated in Figure 2.

The process of bone remodeling is continuous, and the balance between bone resorption and bone formation is maintained through the coordinated activities of osteoclasts and osteoblasts. Depending on the severity of the infection, coccidiosis and necrotic enteritis can induce both acute and chronic inflammation of the gastrointestinal system, which negatively affects the growth and might alter the bone modeling and remodeling of birds [50,69,70]. Previously, it has been observed that during coccidiosis (broilers and laying hen pullets), the number and activity of osteoclasts increased, reducing cortical and trabecular bone volume [8,22,95]. The microstructural architecture of the long bones was measured using X-ray microtomography. The authors found that coccidiosis decreased the bone volume as a fraction of tissue volume (up to 5%), bone mineral content (BMC) (up to 61%), and bone mineral density (BMD) (up to 56%) of both cortical and trabecular bone in infected pullets on 6 and 14 days post-inoculation (DPI) [8,22]. Comparable effects were observed, including decreased BMC, breaking strength, and ash% in broilers infected with *Eimeria* spp. [25,27,28]. Likewise, Sharma et al. (2022) observed a decrease in BMD (up to 35%), BMC (up to 41%), and bone volume (up to 35%) of femurs when laying hen pullets were infected with *Eimeria* spp. The one possible mechanism for this loss in bone properties might be a drastic reduction in feed intake, as observed a couple of days after infection [19,22,95]. However, restriction of feed intake in pair feeding control as that of infected broilers did not have as adverse an effect as that of the infected birds, indicating immunological responses play a significant factor in bone loss. Feed restriction led to a reduction of 10% in BMC, while inflammation resulting from *Eimeria* spp. infection caused an additional 11% loss [8]. It is also interesting to note that while inflammation subsided by 14 DPI, it took longer for the skeletal health of pullets infected with *Eimeria* spp. to recover, requiring up to 28 days post-inoculation, as observed by Sharma et al. 2022.

*Eimeria* infection, alone or combined with the *Clostridium perfringens*, upregulates several cytokines that play a critical role in RANKL expression and osteoclastogenesis. During the infection, naive CD4+ cells differentiated into several subsets, including Th1, Th2, Treg, and Th17 [50,52]. These activated T cells produced several pro-inflammatory and anti-inflammatory cytokines with stimulatory or inhibitory effects on osteoclastogenesis based on the expression of RANKL [4,5,6,92,93,96,97,98,99]. Either alone or together with the *Clostridium perfringens*, *Eimeria* spp. infection upregulates the expression of the pro-inflammatory cytokines (TNF-α, IL-1β, and IL-6) of the innate immune system and APCs, which express the secretion of RANKL [19,50,52]. Furthermore, TNF- α is also a potent inhibitor of osteoblast differentiation by several transcription factors and decreases the phenotype selection of precursor cells to the osteoblast pathway [100]. Among the pro-inflammatory cytokines, IL-17 from Th17 expresses the highest level of RANKL and, has a strong osteoclastogenic effect, was upregulated during the infection. IL-17 further increases local inflammation, and the production of TNF-α and IL-6 further increases the production of RANKL [92]. The production of RANKL by these cytokines activated the RANK signaling pathways in osteoclast precursor cells. It increased the number of osteoclasts (up to 300%), their activity, and elevated bone resorption, as observed previously in chickens by Tompkins et al. 2023 and Sharma et al. 2022.

Furthermore, peripheral APCs (dendritic cells and macrophages) are actively involved in tissue inflammation, and their number rapidly increases during infection. These cells have the capacity to differentiate into osteoclasts, which is governed by cytokine RANKL-RANK expression [101]. Although CD4+ inhibits osteoclastogenesis by increasing the secretion of transforming growth factor-β, while cytokines INF-γ, IL-4, IL-10, and IL-12 by inhibiting the RANK pathways, their functionality is masked by the upregulation of the IL-17, which has strong osteogenic properties [7,92]. On the other hand, IL-17A has been shown to inhibit the wnt signaling pathway, resulting in reduced expression of osteoblast differentiation and early osteocyte markers, thereby inhibiting the osteoblastic differentiation of bone marrow mesenchymal stem cells [102]. The interaction between immune cells and inflammatory cytokines produced during coccidiosis and necrotic enteritis and bone remodeling is illustrated in Table 1. 

## 6. Gut Microbiome and Bone Homeostasis

It has been established that the microbiome helps in nutrient absorption and utilization, regulating the immune response, and reinforcement of the gastrointestinal barrier to prevent microbial translocation [76,108]. The active metabolites from the commensal microbial populations, such as short-chain fatty acids, can stimulate Treg cell activity, which suppresses inflammation and may help reduce bone loss during inflammatory conditions [82,108,109]. However, dysbiosis following coccidiosis or necrotic enteritis might lead to changes in microbial populations that compromise the microbiota’s immunomodulatory functions. Similarly, lactic acid-forming bacteria can help maintain tight junctions in the intestinal epithelial cells, which prevent harmful bacteria from translocating into circulation and bones [110]. Bacterial translocation into bones, as seen in cases such as bacterial chondronecrosis and osteomyelitis in broilers, can initiate inflammatory responses in the femoral head and lead to necrosis. These bacteria further increase the inflammatory responses in the femoral head, leading to necrosis [111,112]. Furthermore, the Gut microbiome plays a significant role in Th17 differentiation. IL-17 from Th17 plays a significant role in RANKL-mediated osteoclastogenesis. Microbial dysbiosis disrupts the equilibrium of the pro-osteoclastogenic pathway and induces osteoclast-mediated bone loss in multiple ways, including the differentiation and inhibition of anti-osteoclastogenic Th1, Th2, and Treg subsets [113].

## 7. Conclusions

In conclusion, we summarized that coccidiosis and necrotic enteritis are two of the most significant gastrointestinal diseases in poultry, with a substantial economic impact on the poultry industry. These diseases affect not only birds’ performance but also their bones’ quality, thereby impacting their overall welfare. The delicate balance between bone-resorbing cells, osteoclasts, and bone-forming cells, osteoblasts, plays a crucial role in bone remodeling, modulated by the immune cells. However, during gastrointestinal infections in chickens, the increased immune response can disrupt this balance through the RANKL-RANK pathway mediated by inflammatory and pro-inflammatory cytokines, ultimately leading to bone loss. Furthermore, microbial dysbiosis further exaggerates bone loss during infection through immune-mediated response or through breaching the gastrointestinal barrier. Although several antibiotic alternatives have been proposed to enhance the immune response against these diseases, increasing the immune response might not always be helpful for maintaining bone quality as some of them might increase osteoclastic activities. Therefore, further studies are necessary to better comprehend the molecular mechanisms of bone loss and their relationship with bone homeostasis in poultry. This understanding will help develop novel therapeutic approaches that selectively improve the immune responses against these diseases without affecting bone homeostasis, ultimately improving chickens’ health, welfare and performance.

## Figures and Tables

**Table 1:** Role of innate immune cells and inflammatory cytokines produced during coccidiosis and necrotic enteritis in osteoimmune system.

Disease	Immune Responses	Cytokines	Effect on Immunity	Bone Resorption	Bone Formation	Possible Mechanism
Coccidiosis Necrotic enteritis	Innate Immunity	Natural killer cells	Antigen recognition and phagocytosis	↑	↓	Osteoclast differentiation, maturation, and activation
	Macrophages Dendritic cells	IL-1β	Proinflammation	↑	↓	Direct activates the RANK signaling to promote osteoclastogenesis [92,93]
		IL-6	Th17 induction	↑	↓	Activation of osteoclastogenesis [99,101,102]
		IL-8	Proinflammation	↑	↓	Osteoclastic activation through RANKL [103]
		IL-12	Proinflammation	↓	↑	Inhibition of RANKL-mediated osteoclast formation [92]
		TNF-α	Proinflammation	↑	↓	Indirect osteoclastic activation through RANKL [97,98,100]
Coccidiosis Necrotic enteritis	Adaptive Immune Response					
	Th1	IFN-γ	Cellular immunity	↓	↑	Inhibit osteoclastogenesis [92]
		IL-2	Proinflammation	↓	↑	Inhibition of RANKL [104]
		IL-12	Proinflammation	↓	↑	Inhibition of RANKL-initiated osteoclastogenesis [92]
		TNF-α	Proinflammation	↑	↓	Indirect osteoclastic activation through RANKL [97,98,100]
	Th2	IL-3	Proinflammation	↓	↑	Blocks RANKL-induced osteoclastogenesis [105]
		IL-4	Humoral immunity	↓	↑	Inhibit osteoclastogenesis, Osteoprotegerin [106]
		IL-9	Antiinflammation	↑	↓	Unknown
		IL-10	Antiinflammation	↓	↑	Suppress bone resorption [92]
		IL-13	Antiinflammation	↓	↑	Inhibit osteoclastogenesis, Osteoprotegerin [106]
	Th17	IL-17	Proinflammation	↑	↓	Induction of RANK and RANKL expression [99,101,102]
		IL-22	Proinflammation	↑	↓	Unknown [99,101,102]
	Tregs	CD4+	Helper T cells	↓	↑	TGF, IL-4, and IL10 [107]
		CD8+	Cytotoxic T cells	↓	↑	Production of OPG [107]

IL-1β: Interleukin 1 β, IL-6: Interleukin 6, IL-8: Interleukin 8, IL-12: Interleukin 12, TNF-α: Tumor necrosis factor α, IFN-γ: Interferon γ, IL-2: Interleukin 2, IL-3: Interleukin 3; IL-4: Interleukin 4, IL-9: Interleukin 9, IL-10: Interleukin 10; IL-13: Interleukin 13, IL-17: Interleukin 17, IL-22: Interleukin 22, RANKL: Receptor activator of nuclear factor κB ligand, RANK: Receptor activator of nuclear factor κB, OPG: Osteoprotegerin, Th1: T helper cells 1 (Cell-mediated immunity), Th2: T helper cells 2 (Humoral immunity), Tregs: Regulatory T cells, Th17: T helper cells 17, ↑ increase, ↓ decrease.

## Data Availability

No new data were generated in this study.
